# Opposing forces and a river into a lake: Relevance to coronary hemodynamics in Kawasaki disease

**DOI:** 10.21542/gcsp.2017.19

**Published:** 2017-10-31

**Authors:** Magdi Yacoub, Heba Aguib

**Affiliations:** 1 Imperial College, London, UK; 2 Aswan Heart Centre, Aswan, Egypt

## Introduction

Kawasaki disease (KD) continues to interest, intrigue, and challenge clinicians, basic scientists, and engineers. One of the defining features of the disease is the development of coronary aneurysms of varying sizes and shapes, reproducing the phenomenon of “River into a lake”, and introducing profound changes in the coronary circulation. These changes reflect many physical, engineering and even cultural beliefs which might be of interest to the readers of this special issue of the Journal dealing with KD. Some of these ideas are presented in this article.

## Opposing forces in the coronary circulation

**Figure 1.**
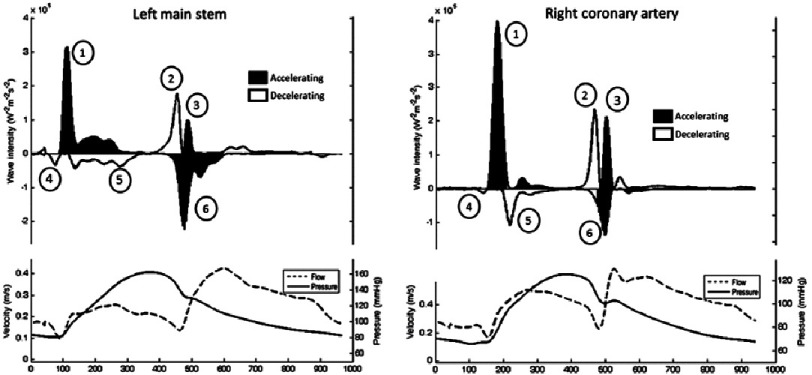
Pressure, flow and separated wave intensity analysis from the left main stem (left) and right coronary artery (right), showing six evident waves per cycle retrieved from WIA, by Broyd et al.^2^

The coronary circulation has many unique features, including timing and regulation^1^. Recent work by Kim Parker, Justin Davies and Chris Broyd, at Imperial College, developed the concept of wave intensity analysis in the coronary circulation^2^. This showed six clearly defined waves, both forward and backward (Figure 1).

## Opposing forces and harmony in Nature

Such analysis shows that, in nature, opposing forces are sometimes necessary to produce harmony. Scale-dependent models have been developed to better understand atmospheric changes using high-performance computers with continuously increasing spatiotemporal resolutions^3^.

**Figure 2.**
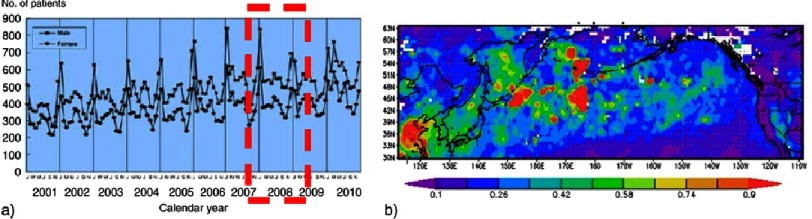
Remote sensing observation of annual dust cycle and possibly causality of Kawasaki disease outbreaks in Japan, seasonality of KD in Japan, by Al-Askary et al. (a) Seasonal Trends of Kawasaki Disease using hospital data (2001–2010). (b) Moderate Resolution Imaging Spectroradiometer (MODIS) Aerosol Optical Depth showing the long range transport of dust from Asia to North America^6^.

Study of opposing forces in “earth science” using computational and observational methods for determining movements of wind and dust, has been valuable in predicting seasonal variations in the incidence of KD^4^ (Figure 2).

**Figure 3.**
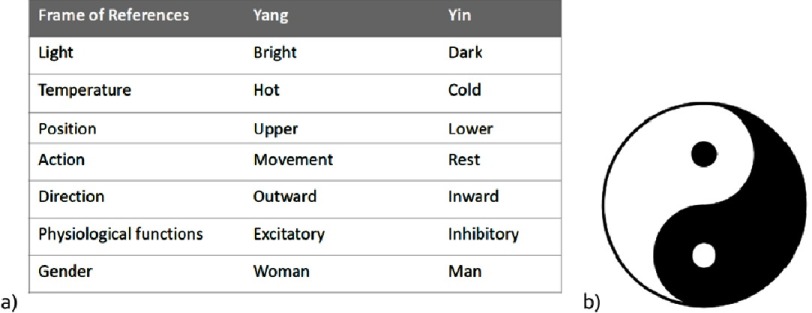
(a) Forces and harmony in Nature, b) The principle of Yin and Yang - opposing pairs of forces - is a fundamental concept in Chinese philosophy, from the 3rd century BCE or earlier.

The most notable opposing pairs of forces are Ying and Yang; light and darkness, respectively (Figure 3). One of the most dramatic opposing forces is the “constant Struggle between the cytoplasm and the nucleus for supremacy” articulated by Sir John Gurdon in his Nobel Prize Acceptance speech^5^. Such thinking was instrumental in evolving the idea of nuclear reprogramming and stem cell biology.

## Asymmetry and blending streams in the circulation

**Figure 4.**
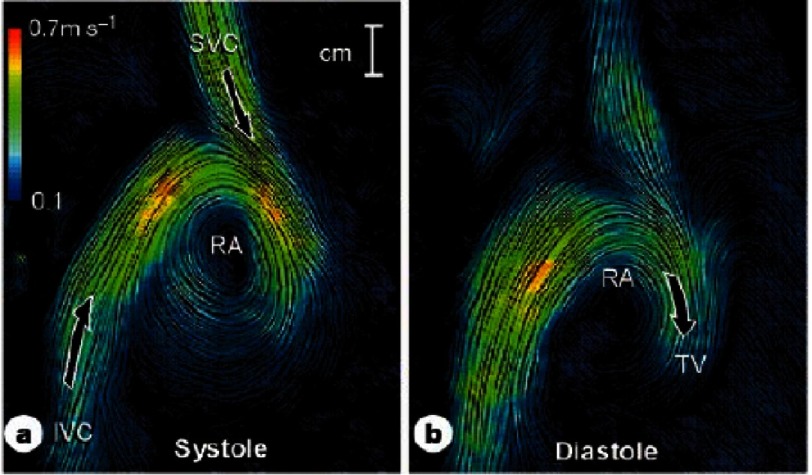
Asymmetry of flow producing harmony: IVC and SVC flow meeting to fill-in the right atrium, by Kilner et al.^7^

**Figure 5.**
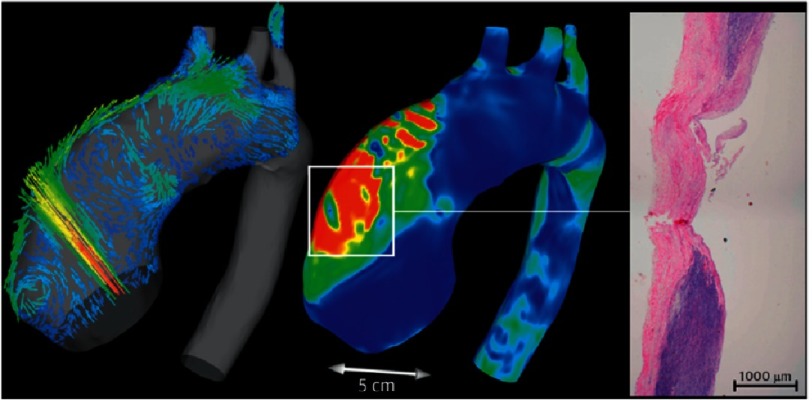
Blood flow and wall shear stress in aortic aneurysm and bicuspid aortic valve, by Torii et al.^8^

The direction of flow from the SVC and the IVC into the right atrium is diagrammatically opposite and elegant way to produce harmony of these two apparently opposing forces is by introducing asymmetry in the anatomic positions of entry of the two vessels into the right atrium^7^ (Figure 4). Visualization of blood flow in vessels using computerized flow dynamics, as well as 4-dimentional MRI studies, are extremely powerful tools to study instantaneous flow in vessels and can be applied in both physiologic and pathologic conditions such as coronary aneurysms in Kawasaki, as well as in larger vessels (Figure 5).

## River into a lake

**Figure 6.**
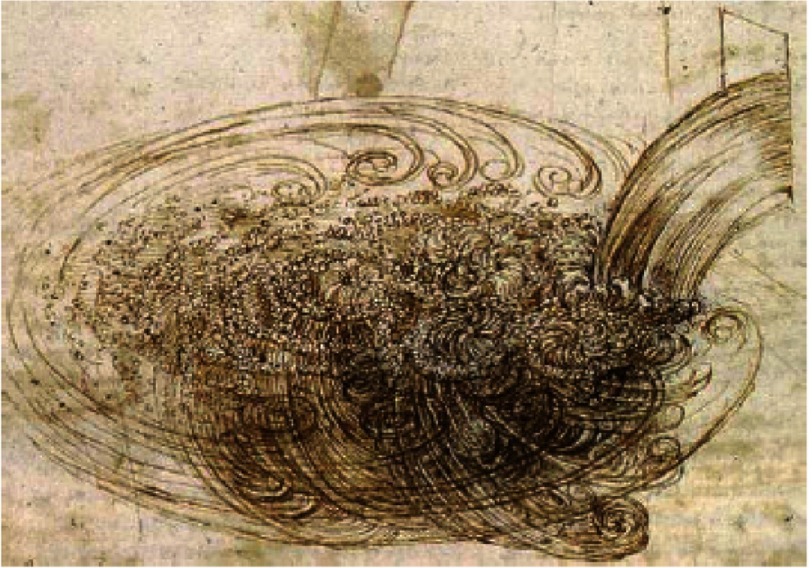
From “Study of water falling into still water”, by da Vinci^12^ and mentioned in [9].

The physical forces operational when a river enters a lake are profound and varied (Figure 6). Such forces have been the subject of many studies including an entire PhD thesis presented to Cambridge University by Hogg^9^.

Another example of the relevance of a river into a lake phenomenon is following the original Fontan operation for tricuspid atresia^10^. This resulted in the distended right atrium ceasing contraction and acting as a “lake” which dissipates kinetic energy and produces clotting. These complications were prevented by the development of the total cavo-pulmonary shunt by Marc De Leval and colleagues^11^.

## Conclusions and future directions

A multidisciplinary approach to the study of the hemodynamics and the structure-function relationship of the lesions in KD is essential for evolving innovative forms of interventional and surgical therapy.
